# Hyperprogression: A Unique Phenomenon of Progression of Existing Tumor Secondary to Immunotherapy

**DOI:** 10.7759/cureus.17992

**Published:** 2021-09-15

**Authors:** Shobha Mandal, Barun Ray, Srijana Baniya Sharma, Joyson Poulose, Vineela Kasireddy

**Affiliations:** 1 Internal Medicine, Guthrie Robert Packer Hospital, Sayre, USA; 2 Internal Medicine, B.P. Koirala Institute of Health Sciences, Dharan, NPL; 3 Urgent Care, Paul Clinic, State College, USA; 4 Hematology and Oncology, Guthrie Robert Packer Hospital, Sayre, USA

**Keywords:** immune checkpoint inhibitor, icis, hyperprogression, pseudoprogression, pembrolizumab

## Abstract

Immunotherapy is a relatively new approach for cancer treatment that has demonstrated prolonged survival by enhancing the body’s immunologic response among advanced cancer patients. Although the benefits of immunotherapy have been well documented, potentially detrimental consequences such as pseudoprogression and hyperprogression have been identified. Hyperprogression is a tumor response in which the existing underlying tumor grows rapidly after initiating treatment with an immune checkpoint inhibitor. This report presents a case of hyperprogression of non-small-cell lung cancer in a 71-year-old male who was initially treated with four cycles of chemotherapy (carboplatin and pemetrexed) and later started on maintenance therapy with pembrolizumab and chemotherapy. Two weeks after receiving the first cycle of immunotherapy, he presented with a complaint of shortness of breath. On repeat computed tomography of the chest, he was found to have a two-fold increase in the size of the preexisting tumor with new large multiloculated right pleural effusion and abdominal ascites.

## Introduction

Immunotherapy has given new hope for different cancer treatments and has been proven to be validated and critically important [1]. It includes various classes of drugs that function as immune checkpoint inhibitors (ICIs) that cease the proliferation of malignant cells. The most widely used ICIs for cancer therapy are cytotoxic T lymphocyte-associated molecule-4 (CTLA-4), programmed cell death receptor-1 (PD-1), and programmed cell death ligand-1 (PD-L1) [2]. ICIs allow T cells to recognize and destroy cancer cells by blocking the inhibitory effects of PD-1 and CTLA-4 on T cells [3]. ICIs are currently used to treat numerous cancers, including melanoma, non-small-cell lung cancer (NSCLC), renal cancer, lymphoma, mismatch repair-deficient colon cancer, etc. [4]. Immune-related adverse events (irAEs) may show different immune responses and can affect any organ. Patients on immunotherapy may develop new response patterns such as hyperprogression and pseudoprogression, which implies a delayed response to the tumor. In patients with lung cancer, if there is any worsening of the pulmonary lesion(s) during ICI treatment, one should consider pseudoprogression, hyperprogression, or interstitial lung disease (ILD) on the list of differentials [5].

This article was previously presented as a meeting abstract at the Chest Conference conducted on October 18-21, 2020.

## Case presentation

**Figure 1:**
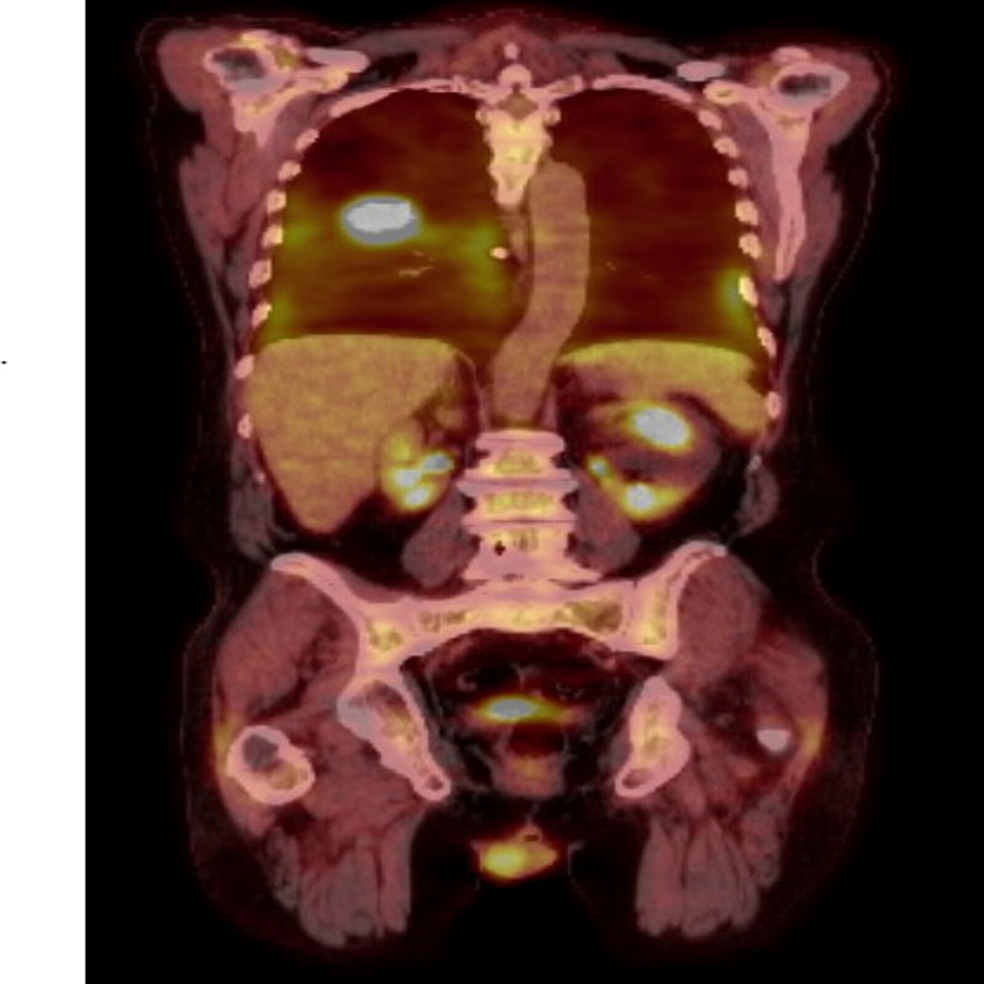
PET-CT scan showing a right perihilar mass with hilar lymphadenopathy. PET-CT: positron emission tomography-computed tomography

A 71-year-old male with a medical history of rheumatoid arthritis (RA) on methotrexate was in his usual state of health one year ago when he started to experience respiratory symptoms. The cough was productive, whitish, and progressively worsening over time. Eight months earlier, he was admitted to the hospital for acute hypoxic respiratory failure. During that admission, computed tomography (CT) of the chest showed a right upper lobe lung nodule along with extensive lymphadenopathy. He underwent a bronchoscopy, but no endobronchial lesion was noted. Biopsies were not performed, and the bronchoalveolar lavage was negative for malignant cells. He underwent a positron emission tomography (PET)-CT scan three months later, which demonstrated a right perihilar mass with hilar lymphadenopathy indicating metastatic disease, with a focal area of increased uptake in the left perihilar region with a maximum standardized uptake value of 3.5 (Figure 1).

**Figure 2:**
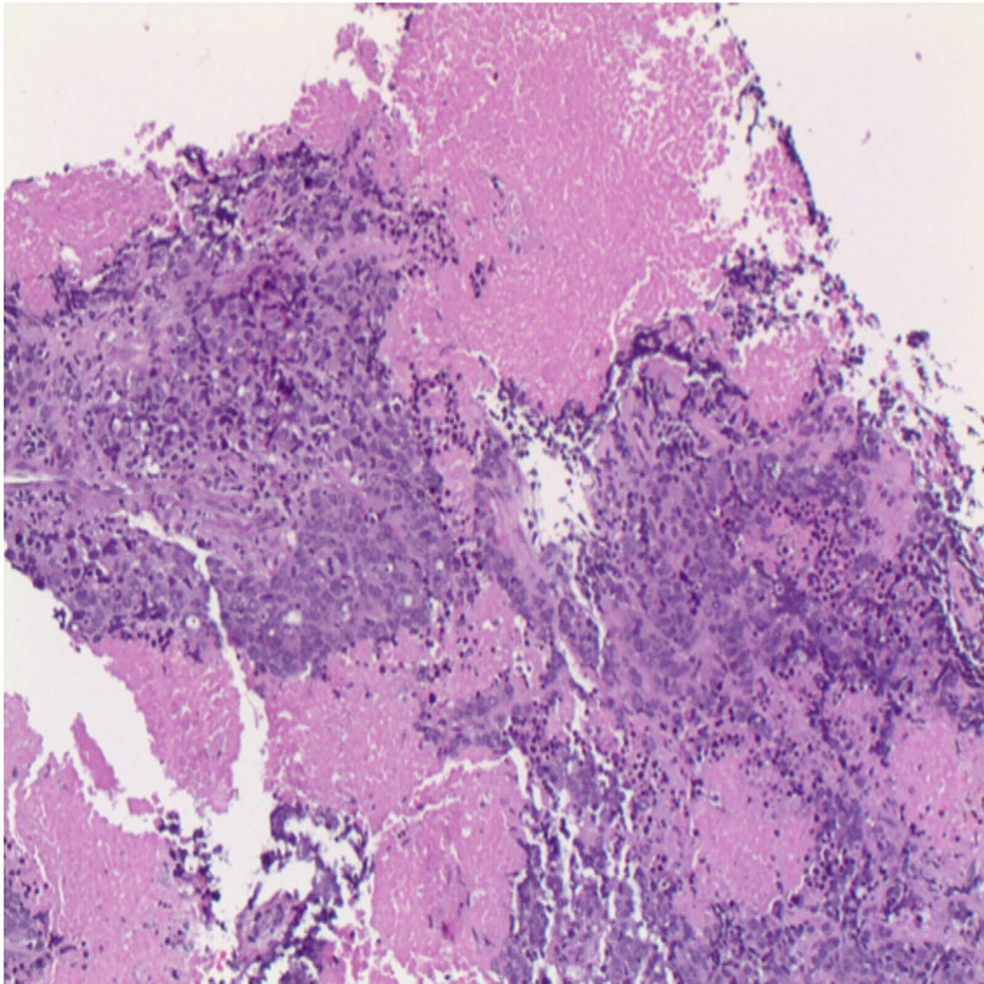
Immunohistochemistry showing poorly differentiated primary adenocarcinoma of the lung (H&E, ×100). H&E: hematoxylin and eosin

**Figure 3:**
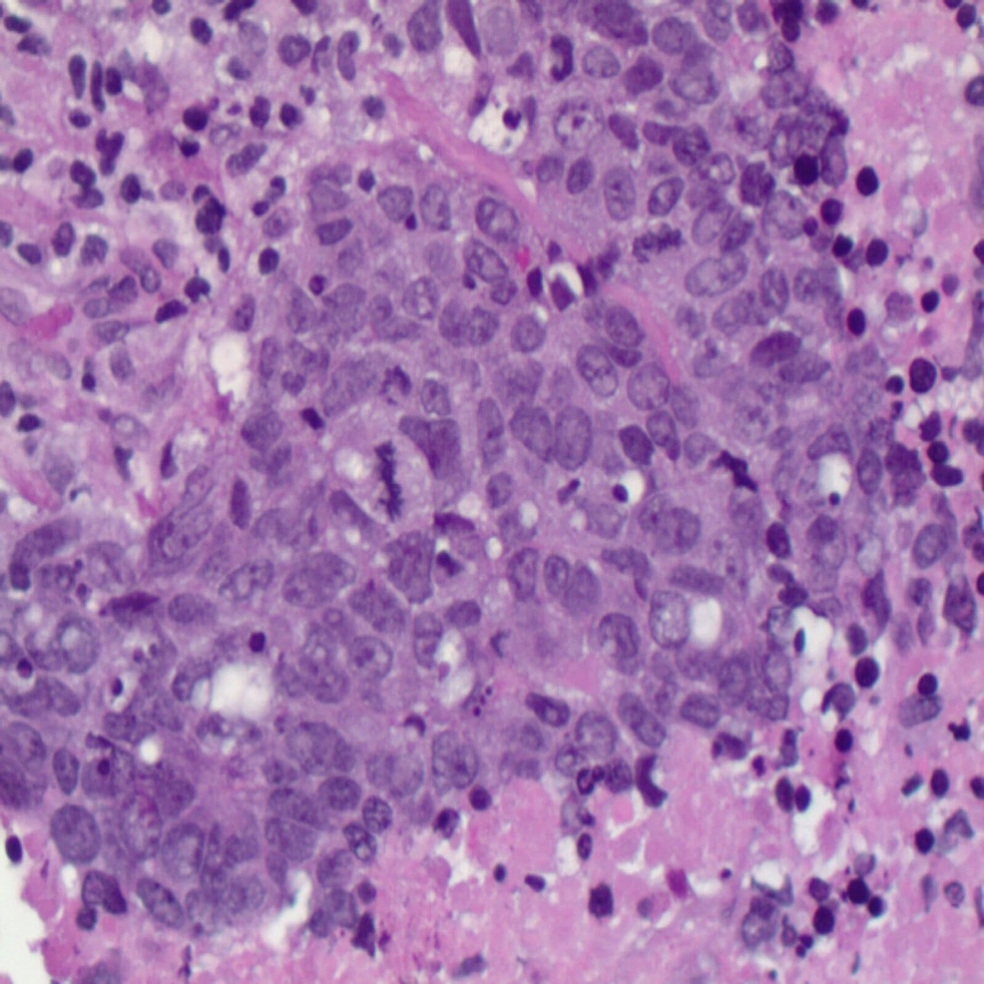
Immunohistochemistry showing poorly differentiated primary adenocarcinoma of the lung (H&E, ×400). H&E: hematoxylin and eosin

**Figure 4:**
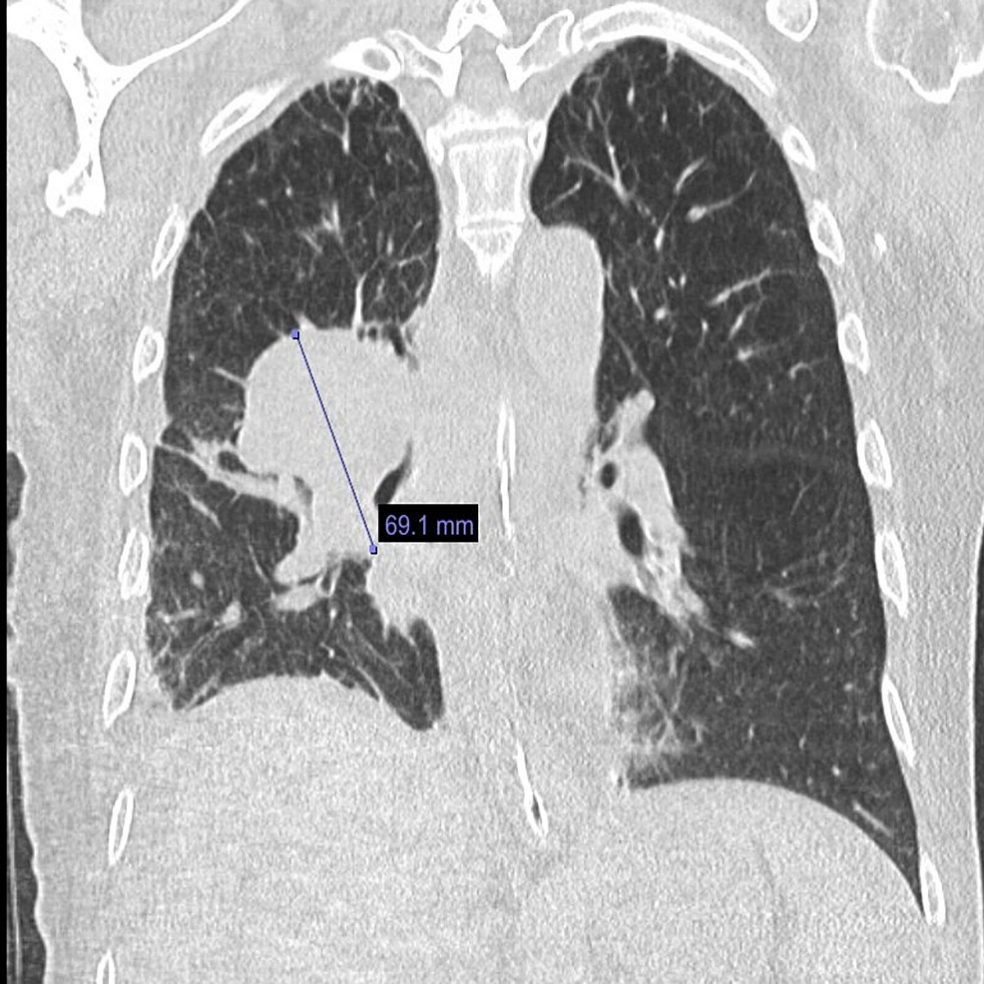
CT scan of the chest showing interval increase in the size of the right lung mass. CT: computed tomography

There was no evidence of distant metastatic disease. Subsequently, he underwent a repeat flexible bronchoscopy with endobronchial ultrasound-guided and transbronchial needle aspiration of the right upper lobe mass and level four and seven lymph nodes, which demonstrated atypical cells, with no evidence of malignancy. He was then referred to surgical oncology and underwent a mediastinoscopy with biopsy of the lymph node, which demonstrated poorly differentiated carcinoma with extensive necrosis. Furthermore, immunohistochemistry was consistent with adenocarcinoma of the lung (Figures 2, 3), with a PD-L1 Tumor Proportion Score of 80%. A repeat CT scan of the chest six months later showed an increase in the right upper lobe lung mass (Figure 4).

**Figure 5:**
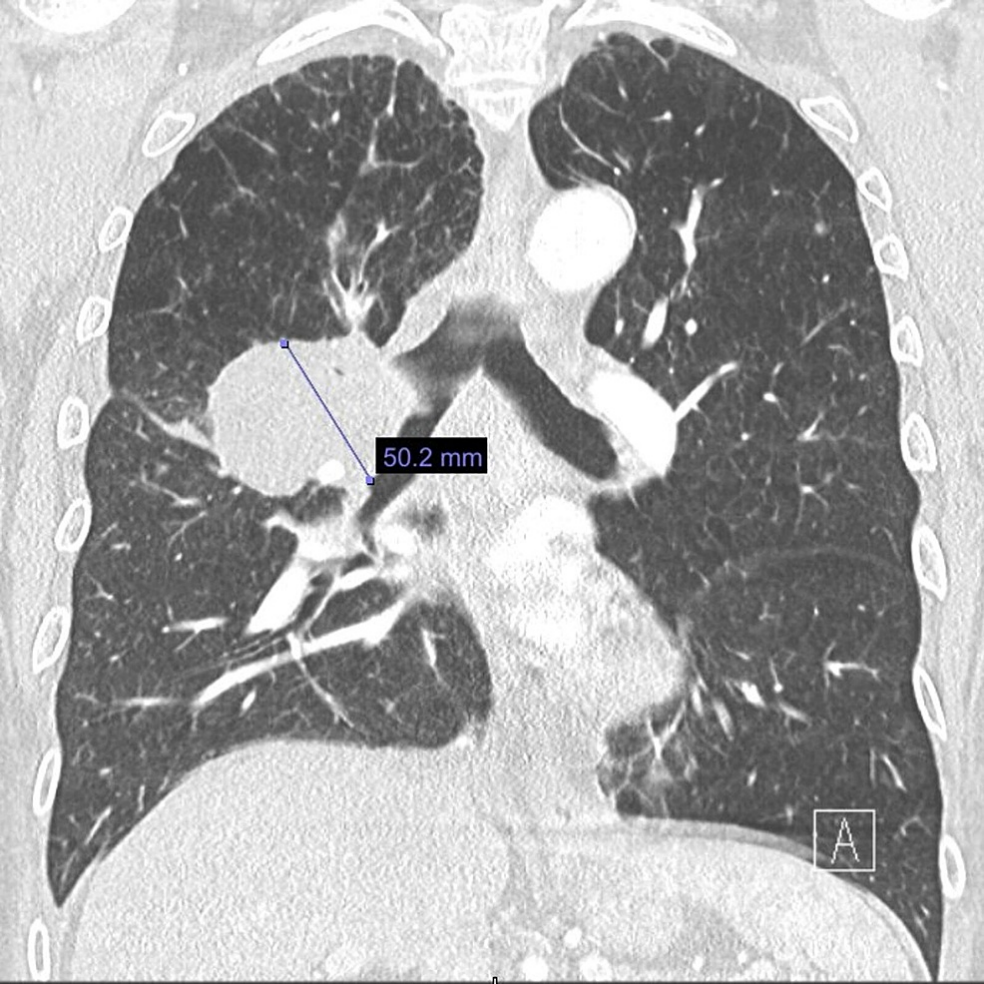
CT scan of the chest post-chemotherapy showing regression of the lung tumor burden. CT: computed tomography

After discussion with the team and the patient, the decision was made to proceed with systemic therapy alone. He initially received “induction” cytotoxic therapy with four cycles of carboplatin AUC5 and pemetrexed 500 mg/m^2^ every three weeks. At first, immunotherapy was overlooked, given the possibility of a flare of his RA. However, after completing four cycles of carboplatin and pemetrexed, his repeat scan showed regression of the lung and liver tumor burden (Figure 5).

**Figure 6:**
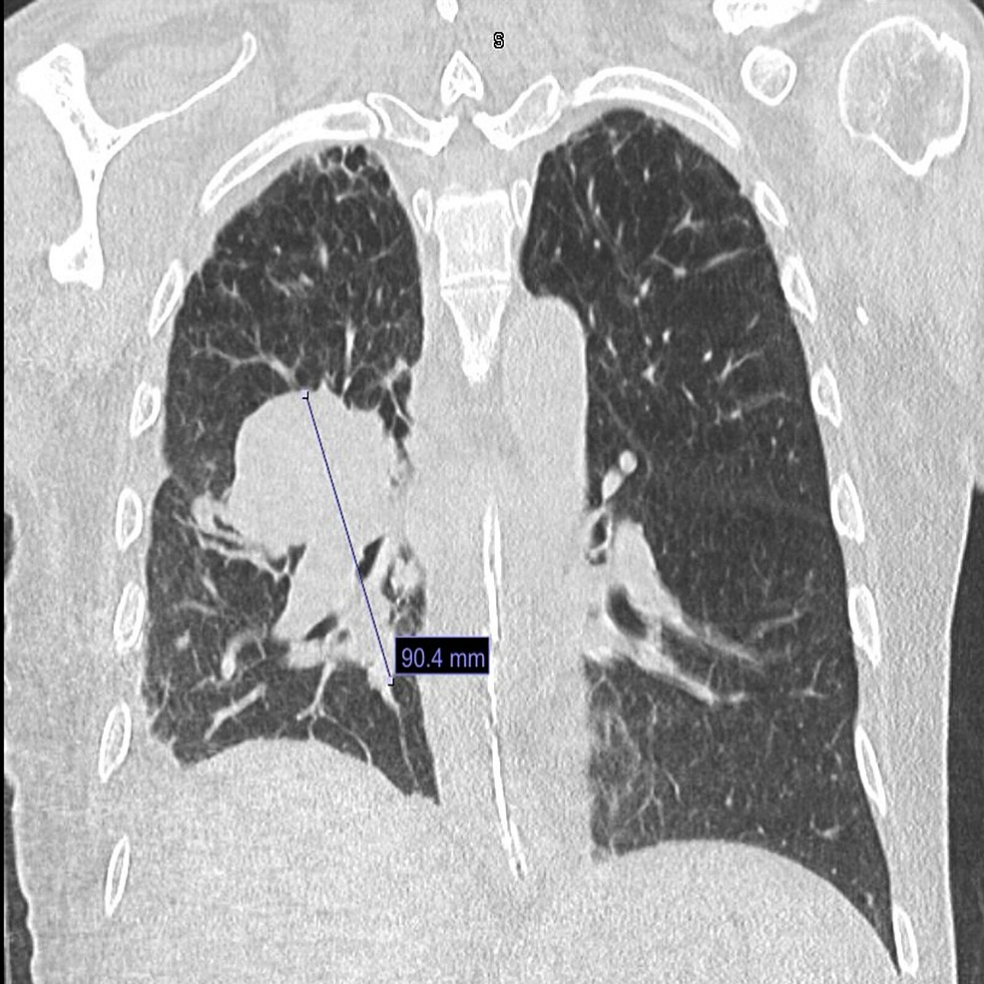
CT chest showing disease progression after immunotherapy with increasing mediastinal lymphadenopathy. CT: computed tomography

Due to his promising clinical response and high PD-L1 (80%), we decided to add immunotherapy with pembrolizumab 200 mg every 21 days to his maintenance chemotherapy. The patient received the first dose of immunotherapy without any complications and was sent home. He returned to the oncology clinic one week later with the complaint of bilateral arm swellings and shortness of breath. Shortness of breath was progressively worsening. He was sent to the emergency department where his oxygen saturation was 87% on room air; hence, he was started on 2 L of oxygen via a nasal cannula. His blood pressure was 87/55 mmHg. Laboratory workup was normal except for lactic acid of 3.9 mmol/L. CT chest pulmonary artery embolism was negative for the pulmonary embolism but showed significant interval disease progression with increased right upper lobe lung mass (Figure 6).

The patient was medically managed with broad-spectrum antibiotics, furosemide, and Solu-Medrol 40 mg twice daily. The patient continued to be managed in the intensive care unit for one week, but his condition worsened. After discussions with the patient and the family regarding his critical illness, prognosis, and the potential need for intubation, the decision was made to proceed only with comfort measures.

## Discussion

Lung cancer is the leading cause of cancer-related death worldwide among men and is the second most common cause of cancer-related death among women. Two significant classes of lung cancer are small-cell lung cancer (SCLC) and NSCLC. NSCLC accounts for approximately 85% of all lung cancers, and is further classified as squamous cell carcinoma, adenocarcinoma, and large-cell carcinoma. SCLC accounts for approximately 15% of lung cancer cases [6]. Both types have differing responses to therapy, different histological findings, and different prognoses post-treatment. Lung cancer treatment is primarily based upon staging. The anatomical extent of the disease is determined by the international TNM-based staging system, where the T classification describes the size and extent of the essential tumor, the N classification depicts the degree of involvement of regional lymph nodes, and the M classification portrays the presence or absence of distant metastatic spread [7]. Treatment options for lung cancer include surgery, radiation therapy, chemotherapy, and targeted therapy. Therapeutic-modality recommendations depend on several factors, including the type and stage of cancer [8]. Apart from traditional treatment, currently, there are two developing strategies regarding using vaccines in the treatment of NSCLC: (1) tumor vaccines and (2) antigen-specific immunotherapy. The goal of vaccine therapy in NSCLC is to shift the immune balance in favor of activation so the host may launch a response to tumor-associated antigens [9,10]. ICIs such as nivolumab, pembrolizumab, and atezolizumab have become essential treatment options for advanced NSCLC.

ICIs were first shown to improve survival in metastatic melanoma followed by NSCLC, leading to the Food and Drug Administration (FDA) approval for ipilimumab (targeting CTLA-4), as well as pembrolizumab and nivolumab (targeting PD-1) for the treatment of the aforementioned solid tumors [11,12]. Clinical trial data have shown that approximately 15-25% of patients with various types of cancer respond to CTLA-4 receptor or PD-1/PD-L1 receptor ICIs [13]. Evidence suggests that the higher the PD-L1 expression by the tumor, the better the response and survival rates with PD-1/PD-L1 ICI treatment [14]. Despite the overall benefits of ICIs for cancer therapy, there are potential life-threatening irAEs related to their use. Patients with NSCLC started on treatment with ICIs may show worsening pulmonary lesions, and pseudoprogression, hyperprogression, or ILD should be kept in the differential diagnoses. Nearly 8-14% of NSCLCs treated with ICIs are found to have hyperprogressive disease [15]. The Response Evaluation Criteria in Solid Tumors (RECIST) standards were published in 2000 by an international collective to define the response of tumors during treatment. Depending on the response criteria of a tumor, it was labeled progressive disease if there was at least a 20% increase in tumor size compared to pre-treatment size or if there was the appearance of one or more new lesions. The RECIST criteria were not found equally effective in evaluating immunotherapy on tumor response; hence, immune response-related criteria were developed in 2009 [16].

Hyperprogression was defined multiple times depending on the findings of the clinical trial. Champiat defined hyperprogression as RECIST progression at the first evaluation and at least two-fold tumor growth rate (TGR) increase between pre-immunotherapy and immunotherapy period [16]. Ferrara et al. compared pre- and post-immunotherapy TGR in a retrospective cohort of 406 patients with advanced NSCLC treated with PD-1/PD-L1 inhibitors, defining hyperprogressive disease as RECIST progression at the first evaluation and a difference between on-treatment and pre-treatment TGR (∆TGR) exceeding 50% [17,18]. Patients should be evaluated with repeat imaging every three to four weeks to examine the response to immunotherapy. In patients developing hyperprogression, immunotherapy treatment should be stopped and the patient should be managed appropriately. Similar to hyperprogression, another phenomenon known as pseudoprogression is a radiological finding in which the tumor burden or the number of tumor lesions increases initially and then decreases over time. In this phenomenon, patients treated with immunotherapy experience an initial increase in tumor burden through enlargement of target lesions and/or development of new lesions, followed by a subsequent decrease in the tumor burden qualifying as a partial or complete response [19,20].

## Conclusions

Immunotherapy offers new hope in cancer treatment and is slowly getting FDA approval for the treatment of multiple malignancies which have failed other treatments. As the treatment is evolving, we are noting different side effects of the treatment. Among the different side effects, hyperprogression and pseudoprogression are new phenomena. Our case emphasizes that physicians need to be aware of new tumor response patterns to ICIs such as hyperprogression and pseudoprogression to manage patients appropriately. These side effects can be fatal if not treated timely.
